# Extraction, Characterization, and Structure of a Novel Heteropolysaccharide from Lepidium sativum and Its Effects on Wound Healing in Diabetic Rats

**DOI:** 10.1155/2022/7858865

**Published:** 2022-08-23

**Authors:** Sirine Ben Slima, Naourez Ktari, Aicha Chouikhi, Amina Hzami, Sana Bardaa, Imen Trabelsi, Basma Ben Salah, Riadh Ben Salah

**Affiliations:** ^1^Laboratory of Biotechnology Microbial Enzymatic and Biomolecules (LBMEB), Center of Biotechnology of Sfax, University of Sfax, 3018 Sfax, Tunisia; ^2^Laboratory of Enzyme Engineering and Microbiology, National School of Engineering of Sfax (ENIS), University of Sfax, 1173-3038 Sfax, Tunisia; ^3^Department of Life Sciences, Faculty of Science of Gabes, University of Gabes, Omar Ibn Khattab Street, Gabes 6029, Tunisia; ^4^Pharamcology Laboratory, Faculty of Medicine of Sfax, University of Sfax, Majida Boulila Road, 3028 Sfax, Tunisia; ^5^Faculty of Medicine of Sfax, University of Sfax, Majida Boulila Road, 3028 Sfax, Tunisia

## Abstract

The present study undertakes the extraction of a novel polysaccharide from Lepidium sativum (PLS) and the determination of its physicochemical composition and antioxidant properties, as well as its potential wound healing activity in alloxan-induced diabetic rats. This polysaccharide presented a lighter natural color, whose luminosity (*L*∗), red-green intensity (*a*∗), and blue-yellow intensity (*b*∗) were recorded at 63.26, 5.87, and 27.28, respectively. The PLS was structurally characterized by Fourier transform infrared (FT-IR) spectroscopy, UV spectrum, high performance liquid chromatography (HPLC), gas chromatography (GC), nuclear resonance magnetic (NMR), and high-pressure gel filtration chromatography. The FT-IR and UV spectra showed the characteristic band of polysaccharides. According to HPLC, the crude PLS is a heteropolysaccharide composed of glucose, xylose, and galactose. Results obtained by ^1^H NMR indicated that PLS consisted of three monosaccharide residues with *α* and *β* anomers. This novel polysaccharide had an average molecular weight of 98.51 kDa and displayed potential antioxidant activities determined through three different assays: scavenging activity against 2,2′-azino-bis-3-ethylbenzothiazoline-6-sulphonic acid (ABTS), 2,2-diphenyl-1-picrylhydrazyl (DPPH) scavenging assay, and reducing power. These results strongly support the beneficial effects of the PLS to accelerate wound healing in diabetic rats. Indeed, its application significantly increased wound contraction percentage (98 ± 1.11%) after 14 days of experiment. Furthermore, the histological assessment of the PLS-treated group demonstrated complete reepithelialized wounds by accelerating collagen synthesis. In general, the findings affirmed that PLS is efficient on wound closure in alloxan-induced diabetic rats.

## 1. Introduction

Wounds result in a simple or severe damage to the function and anatomical structure of an organ, such as the skin or other tissues [1, 2]. The healing of cutaneous wounds can be characterized by four phases, including hemostasis, inflammation, proliferation, and remodeling. However, chronic wounds that fail to develop satisfactorily through the normal phases of healing represent a major complication to the healthcare systems [3]. They are generally characterized by nonmigratory epidermis, inflammation excess, decreased bioactive growth factor levels, and impaired fibroblast growth [4–7]. These problems are related to infection, obesity, immunosuppression, and diabetes (such as vasculopathies and neuropathies) [8], which contribute to the formation of diabetic ulcers. The cutaneous wound healing in acute diabetic patients is related to delayed and abnormal inflammatory response, which is characterized by the deficiencies in the neutrophils number in the wound site. This presents a major source of the increase of bacterial infection that drives to a chronic nonhealing wound state which can cause a significant healthcare burden and sometimes necessitates hospitalization and amputation. Due to the lack of control and preventive measures, the incidence of an impaired healing process in diabetic patients is constantly increasing [9].

Recently, there has been growing interest in the development of the chronic wound dressing agents that can effectively protect from damage, decrease complications, and accelerate lesion healing in diabetic wound healing using experimental animals [1, 10].

Natural biomaterials have received increased attention for medicine treatment, most particularly tissue engineering due to various good properties, safety, low cost, and high biocompatible nature [6, 11, 12]. Natural bioactive polysaccharides purified from plants are superior to other polymers for their biological activities and various chemical and physical properties [10, 13], exhibiting beneficial effects on human skin cells. In fact, not only do polysaccharides stimulate the complement system, fibroblasts, keratinocytes, vascular endothelial, and polymorphonuclear cells, but they also modulate the inflammatory phase [1, 6]. A study was carried out on the model of a diabetic rat induced by alloxan to test the wound healing effect of the polysaccharide in vivo. This method was used to study and screen new drugs and new therapeutic modalities [14].

Lepidium sativum, also known as garden cress, is a fast-growing annual herb of the Cruciferae family, which is cultivated throughout the world as a culinary vegetable. It grows throughout the world, widely in the Middle East, USA, and Europe [15]. This herbal medicine has been revealed effective against various ailments, including hypertension, inflammation, arthritis, bronchitis, hepatotoxicity, cancer, and diabetes [16].

L. sativum seed application has been established in ancient India and the Middle East countries for the treatment of various diseases in traditional medicine [17]. They contain volatile aromatic oil and different components, namely, vitamins, proteins, carbohydrates, fatty acids, phosphate, calcium, and trace elements [18–20].

The present study is aimed at exploring the characterization and antioxidant activities of a novel polysaccharide from L. sativum (PLS), and for the first time, the effects of PLS on chronic wound healing in diabetic rat models.

## 2. Material and Methods

### 2.1. Reagents

All the chemicals were obtained from Sigma Chemical Co. (St. Louis, MO, USA). Dry L. sativum seeds were purchased from a local market in Sfax city, Tunisia. For chronic wound healing, “Cytol Centella” was used as a reference cream that was purchased from local drugstore.

### 2.2. Preparation of L. sativum Polysaccharide

The powder obtained after crushing seeds was preextracted with ethanol. The residue was then exhaustively extracted with distilled water (90°C) and filtered [21]. After the evaporation of filtrates, the concentrated liquid was precipitated by ethanol addition during 24 h at 4°C and then centrifuged (4500 rpm, 15 min) using centrifuge (Hettich Zentrifugen, ROTINA 380R, Germany). Then, the precipitate was lyophilized to obtain PLS powder. The yield of the crude PLS was calculated based on the wet weight of cress seed powder.

### 2.3. Physicochemical Characteristics of PLS

The ash and moisture contents were determined according to the Association of Official Analytical Chemists (AOAC) standard methods [22]. While the total crude lipid was determined by gravimetric method after the Soxhlet extraction of samples [21], the crude protein was identified by the factor of 6.25 after multiplying total nitrogen content. The phenol-sulfuric-acid method was used to determine the total carbohydrate content [21].

The pH analysis of PLS (1%) was evaluated using a digital pH meter (Systronics Instruments, India). The color of PLS was determined using a Color Flex spectrocolorimeter (Hunter Associates Laboratory Inc., Reston, VA, USA) and reported as luminosity (*L*∗), red-green intensity (*a*∗), and blue-yellow intensity (*b*∗).

### 2.4. Spectroscopic Analysis

#### 2.4.1. UV Absorption Peak Detection

The UV absorption spectra of the PLS at concentration 0.01% was recorded in the wavelength range from 200 to 800 nm [23].

#### 2.4.2. FT-IR Spectrometric Analysis

The absorption spectra of PLS were obtained using a Nicolet FT-IR spectrometer. All spectra were scanned in the range of 4000-500 cm^−1^. The OPUS 3.0 data collection software program (Bruker, Ettlingen, Germany) was used to analyze the obtained data.

#### 2.4.3. NMR Analysis

Spectroscopy NMR experiments of PLS were recorded on a Bruker 400 spectrometer (Bruker Biospin AG, Fallanden, Switzerland) at 25°C. The 20 mg of powdered samples was dissolved in 1 mL deuterated water (D_2_O). ^1^H NMR spectra were recorded at a frequency of 400 MHz. The chemical shift was expressed in parts per million.

#### 2.4.4. High-Pressure Gel Filtration Chromatograph

To determine the average molecular weight, we utilized the high-pressure gel filtration chromatograph employing a gel filtration high-pressure chromatography equipped with a refractive index detector using Zorbax PSM 300 column (62∗250) with a mobile phase of bidistilled water, a flow rate of 0.8 mL/min and temperature 30°C [21]. The average molecular weight (Mw) of the PLS was evaluated by its retention time in comparison with the calibration curve using different known molecular weights of dextran.

#### 2.4.5. Determination of PLS Composition

The determination of the monosaccharide compositions was carried out by HPLC. Briefly, PLS was mixed with 250 *μ*L of trifluoroacetic acid (TFA, 4 M), and the obtained mixture was incubated at 100°C during 8 h of hydrolysis.

The HPLC was performed using an Aminex HPX-87H column with a mobile phase of H_2_SO_4_ (0.001 N), column temperature of 60°C, and a flow rate of 0.4 mL/min.

Monosaccharide composition of polysaccharides was also determined by GC. In fact, the Agilent gas chromatography system (Agilent 19091S-433 Agilent Technologies, USA) was used according to the method of Luo et al. with some modifications [24]. Briefly, the hydrolyzed products were acetylated with N,O-Bis(trimethylsilyl)trifluoroacetamide (BSTFA) and pyridine for 1 h at 60°C. According to the nature of the compound, the injection volume was 1 *μ*L, the mode used was spitless, and the carrier gas was high-purity helium. The temperature was maintained at 150°C for 2.0 min, and the temperature was programmed to 250°C at 3°C/min and then maintained at the same temperature for 3 min.

### 2.5. In Vitro Antioxidant Activities of PLS

#### 2.5.1. DPPH Scavenging Assay

The DPPH radical scavenging activity of PLS was evaluated as previously described [21]. Briefly, 125 *μ*l of DPPH (0.02%) in ethanol solution was prepared and then mixed with 500 *μ*L of the samples of various concentrations (1 to 3 mg/mL) and 375 *μ*L of ethanol. The mixture was shaken and left in the dark at 25°C for 1 h before the absorbance at 517 nm was measured. Butylated hydroxytoluene (BHT) was used as a positive control.

#### 2.5.2. ABTS^+^ Radical Scavenging Activity

The ABTS^+^ radical scavenging activity of PLS was realized as described by Ben Slima et al. [21]. The ABTS radical monocation was the result of the reaction between 7 mM ABTS (2,2′-azino-bis (3-ethylbenzothiazoline-6- sulphonic acid) reagent in H_2_O and 2.45 mM K_2_S_2_O_8_ (potassium persulfate) stored in the dark at room temperature for 12 h. The sample (0.5 mL) was mixed with 1 mL ABTS radical and then kept in obscurity for 6 min at room temperature. The absorbance of the mixture was measured spectrophotometrically at 734 nm.

#### 2.5.3. Reducing Power Determination

The amount of 0.5 mL of the sample solutions at different concentrations (1-3 mg/mL) was mixed with 1.25 mL phosphate buffer (pH 6.6; 0.2 M) and 1.25 mL of potassium ferricyanide solution (10 g/L). The mixtures were then incubated at 50°C for 30 min. The amount of 1.25 mL of trichloroacetic acid (TCA, 10%) was added to stop the reaction. The quantity of 1 mL aliquot of the supernatant from each sample was mixed with 1 mL of distilled water and 0.25 mL of ferric chloride solution (0.1%). After 10 min reaction incubation, the absorbance was measured at 700 nm [6]. Indeed, this method is based on the reduction of ferric (Fe^3+^) to ferrous (Fe^2+^), which is accomplished in the presence of antioxidants. Substances having a reduction potential react with potassium ferricyanide and form potassium ferrocyanide which further reacts with ferric chloride (FeCl_3_) to form an intense Prussian blue complex having a maximum absorbance at 700 nm [25].

### 2.6. In Vivo Assays of the PLS Effect on Wound Healing in a Diabetic Rat Models

PLS was dissolved at a final concentration of 15 mg/mL in distilled water and in 30% of glycerol solution with stirring until hydrogel PLS was obtained.

#### 2.6.1. Animals and Experimental Designs

Experimental diabetes was induced in animals by intraperitoneal injections of alloxan at a dose of 125 mg/kg. Diabetes onset was confirmed by the classic symptoms (polyuria, polydipsia, and polyphagia) and hyperglycemia, detected by reactive strips.

Diabetic animals were divided into four groups: control diabetic group treated with a saline solution (group I), diabetic group treated with glycerol (group II), positive group in which animals were treated with Cytol Centella cream (group III) and diabetic group treated with PLS hydrogel (group IV). The animals were treated at 1, 3, 5, 10, 12, and 14 days postwounding. Three independent experiments on three animals per group were used. After being anaesthetized, a circular (1.5 cm diameter) full-thickness defect was created in animals using surgical scissors. During the first two days, the wound areas were covered with sterile dressing to avoid the manipulation of the wound. After 13 days of treatment, the wound tissue was excised to be used in hydroxyproline content and histological studies.

#### 2.6.2. Qualitative Assessment of Wound Healing

Wound closure was assessed by photography using Panasonic LUMIX digital camera (LUMIX FZ1000 4 K QFHD/HD 16X) attributing the wound color code as follows: bright red = blood in the wound; dark red = blood coagulation, red = granulation tissue, and pink = epithelialization phase [26].

#### 2.6.3. Measurement of Wound Area and Wound Contraction Rate

The wound boundaries were traced using a transparent paper. For design and drafting, the Autodesk AutoCAD 2015 software application was used. The wound contraction rate was calculated as follows:

(1) $$\begin{array}{c} \text{Wound contraction rate }\left(\%\right)=\frac{A_{0}-A_{d}}{A_{0}}\times 100, \end{array}$$

where *A*_0_ is the initial wound size, and *A*_*d*_ is wound size on a given day.

#### 2.6.4. Histologic Examination

The wound tissues were placed in neutral-buffered formalin solution (10%) and embedded in paraffin wax. Following incorporation in paraffin and cutting, 5 *μ*m thick sections were stained with hematoxylin–eosin to observe by a light microscope (Olympus CX41, Tokyo, Japan).

#### 2.6.5. Hydroxyproline Estimation

The hydroxyproline contents of rat tissue samples were estimated according to the method of Trabelsi et al. [1]. Wound tissues were dried in a hot air oven at 60-70°C to a constant weight and then hydrolyzed with 6 N HCl for 4 h at 150°C. The hydrolysates were neutralized to pH 7.0 and then subjected to chloramine-T oxidation for 20 min. The reactions were terminated by the addition of 0.4 M perchloric acid, and the color developed with the help of Ehrlich reagent at 60°C. The absorbance was measured at 650 nm. Hydroxyproline concentrations were presented as mg/g of dry tissue weight.

### 2.7. Statistical Analysis

Data were expressed as mean ± standard deviation (SD). The results were statistically analyzed with the SPSS program (V21.0) using the one-way variance analysis (ANOVA) procedure followed by Tukey's post hoc test. When *p* < 0.05, differences were considered significant.

## 3. Results and Discussion

### 3.1. Physicochemical Characteristics of Crude PLS

The chemical analysis has demonstrated that PLS is composed primarily of carbohydrates (88.58%) and displayed low protein (1.23%) and lipid (0.03%) contents (Table 1). The high sugar content can be influenced by the extraction method efficiency. The most interesting components of PLS value were similar for polysaccharides extracted from Sorghum bicolor [21] and Trigonella foenum-graecum, recording 78.85 and 92.4%, respectively [11]. The low lipid and protein contents imply their efficient elimination from the vegetal material. Moreover, protein contents in the polysaccharides that were extracted from vegetables presented low quantities of protein, which agrees well with our results [1, 27].

As shown in Table 1, the PLS extraction yield was 8.2 ± 0.99%. This value was better than that of other vegetable polysaccharides such as adzuki beans (6.31%) [28] and chickpea (5.56%) [29]. Such differences could be affected by the ratio of water to raw material, time, and temperature [21].

PLS presented light color whose *L*∗ value was 63.26. The *a*∗ and *b*∗ values were recorded at 5.87 and 27.28, respectively (Table 1). Similar results were obtained by Trabelsi et al. [30] who reported that crude polysaccharide extracted from katan presented the light-yellow color. This characteristic enhanced their suitability to be incorporated in nonfood formulations. Besides, the pH of 1% PLS solution at 25°C was recorded at 7.2.

### 3.2. Spectroscopic Analysis

#### 3.2.1. UV Absorption Peak Detection

**Figure 1:**
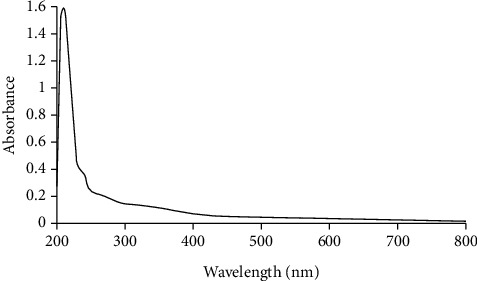
Scan of PLS within the wavelength range of 200-800 nm.

PLS was recorded on 200 to 800 nm range in UV-visible spectrum. As shown in Figure 1, UV-visible absorbance variations revealed a maximum absorption peak at 210 nm. The different levels of absorbance peaks ranging from 200 to 220 nm confirmed the presence of polysaccharides [21].

#### 3.2.2. FT-IR Spectrometric Analysis

**Figure 2:**
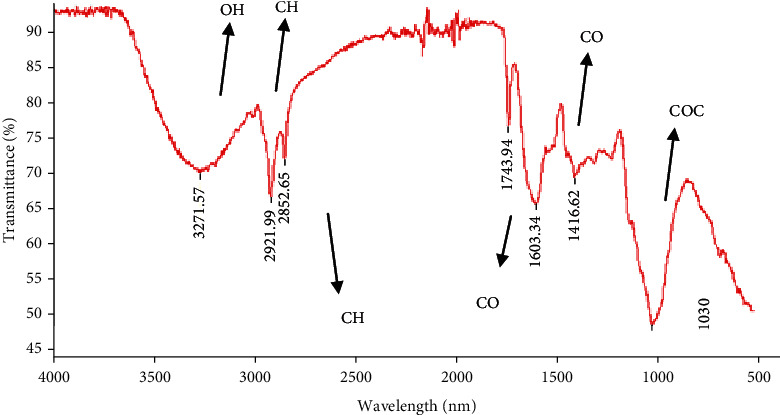
FT-IR spectra of PLS.

The characterization of the structure and the functional groups of polysaccharides was conducted via FT-IR spectroscopy. As presented in Figure 2, PLS displayed typical peaks at 3271.57, 2921.99, 2852.65, 1743.94, 1603.34, 1416.62, and 1030 cm^−1^.

PLS broadly exhibited OH stretching vibration at 271 cm^−1^, indicating the formation of intermolecular and intramolecular hydrogen bonds. The bands at 2921.99 cm^−1^ may be assigned to the stretching vibrations of CH. An additional band observed at 1603.34 cm^−1^ can be correlated to the bound water [21]. The absorbance at 1401 cm^−1^ is characteristic of the CO group. The region between 1138 cm^−1^ and 1022 cm^−1^ suggests that the skeletal modes of the polysaccharide are pyranose rings [31]. Weak absorption below 1000 cm^−1^ reported the visible bands presence and/or possible linkages between the molecules of monosaccharide [32, 33].

#### 3.2.3. NMR Analysis

**Figure 3:**
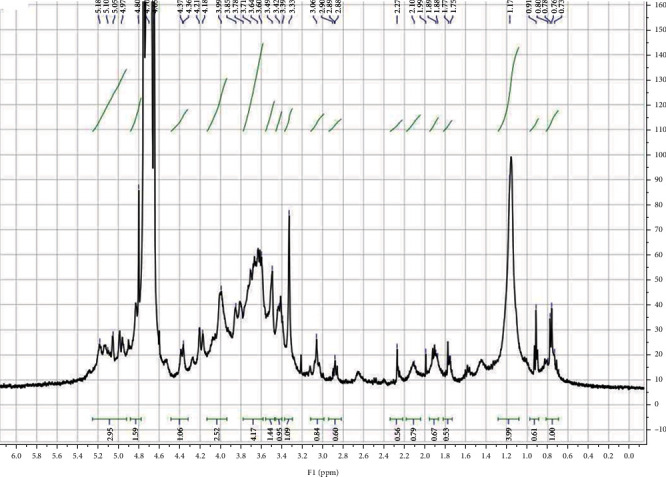
^1^H NMR spectra of PLS.

In the ^1^H NMR spectrogram (Figure 3), a narrow region in the range of 3-5 ppm reveals several similar sugar residues which confirm the presence of polysaccharides [6]. Generally, the signals less than 4.0 ppm corresponded to *β*-anomeric proton while the chemical shift values of *α*-anomeric protons were mostly larger than 4.0 ppm [30]. According to relevant literature data, the different doublet peaks above 5 ppm characterized xylose, glucose, and galactose [34]. In fact, signals found at 4.97, 5.05, and 5.10 correspond to xylose, glucose, and galactose contained in PLS, respectively. The most important region for predicting galactose is around 3.99 ppm. Also, the multiplets around 3.7 ppm, an intense peak at 3.49, and the triplet around 3.39 ppm identify galactose, glucose, and xylose, in PLS, respectively [34]. An intense peak signal observed at 1.17 ppm identifies the carbon group (R-CH2-CH3) [30]. Therefore, NMR of PLS presented the signals of region of three monosaccharides (xylose, galactose, and glucose).

#### 3.2.4. High-Pressure Gel Filtration Chromatograph

**Figure 4:**
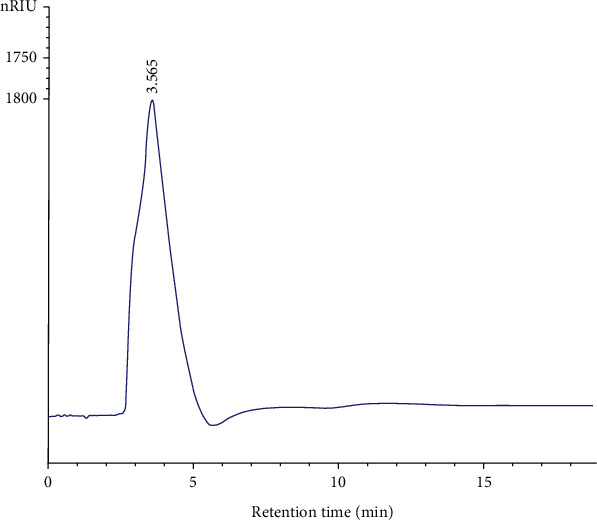
Gel filtration chromatographs of PLS.

As shown in Figure 4, the gel filtration chromatograph recorded one major peak at 3.565 min. Their average molecular weights were estimated to be 98.51 kDa based on the regression equation. Similar results were obtained by Bayar et al. [35] who reported that the polysaccharide extracted from cladodes molar mass was around 93.59 kDa.

#### 3.2.5. Monosaccharide Composition Analysis by HPLC

**Figure 5:**
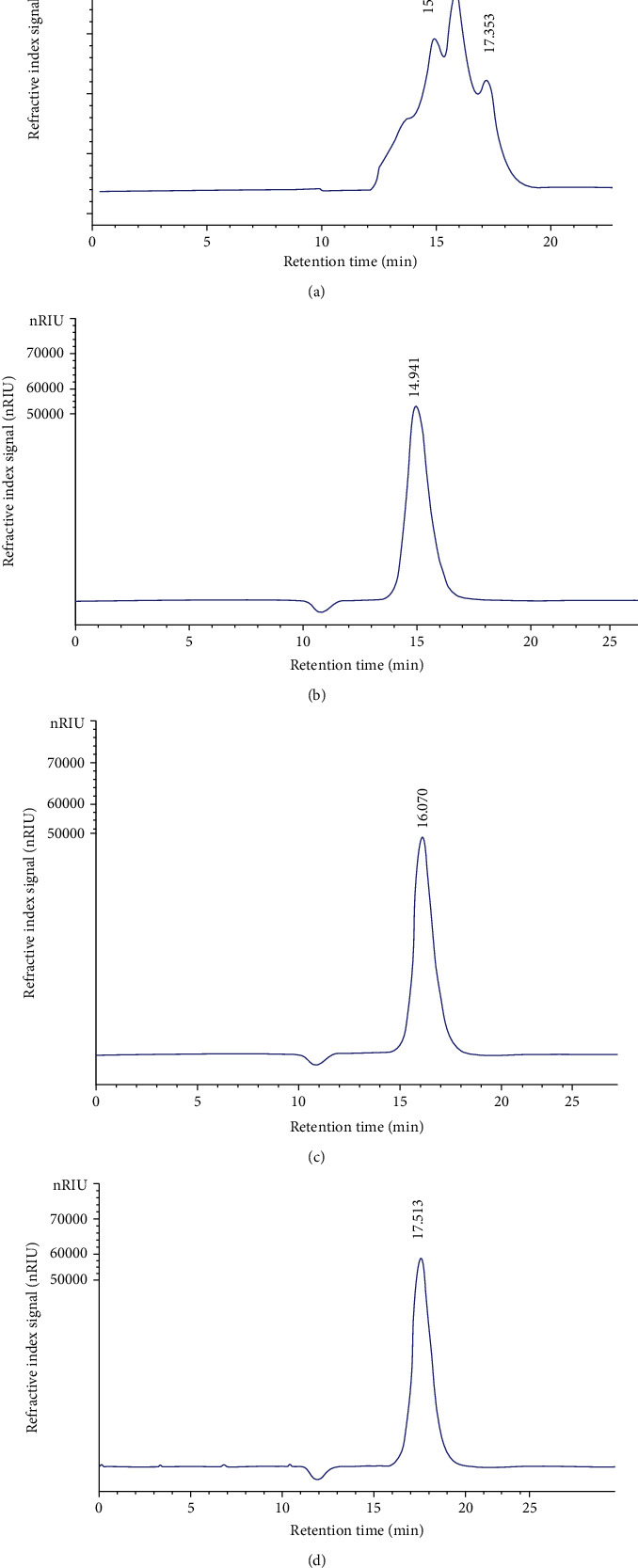
HPLC analysis of PLS (a). HPLC analysis of glucose (b). HPLC analysis of galactose (c). HPLC analysis of xylose (d).

As indicated in Figure 5, the PLS is composed of glucose (15.101 min), galactose (16.014 min), and xylose (17.353 min). In a previous study, it has been reported that cress seed is a combination of uronic acid units, L-arabinose, L-rhamnose, D-galactose, D-galacturonic acid, and D-glucose [36]. The variability of polysaccharides composition may be linked to exogenous parameters as nutriment, temperature, and water concentration [37].

#### 3.2.6. Monosaccharide Composition Analysis by GC

**Figure 6:**
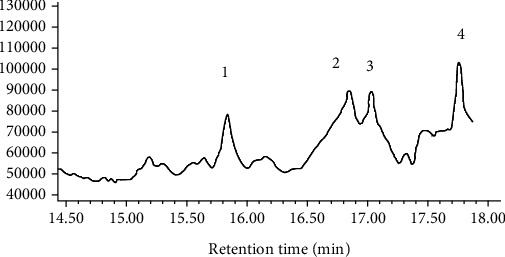
GC of PLS.

PLS were hydrolyzed and acetylated for further analysis by GC. Interestingly, as shown in Figure 6, PLS were composed of glucofuranose (15.838 min), D-xylose (16.710 min), *β*-D-galactofuranose (16.853 min), and D-galactose (17.767 min).

### 3.3. In Vitro Antioxidant Activities of PLS

#### 3.3.1. DPPH Scavenging Assay

**Figure 7:**
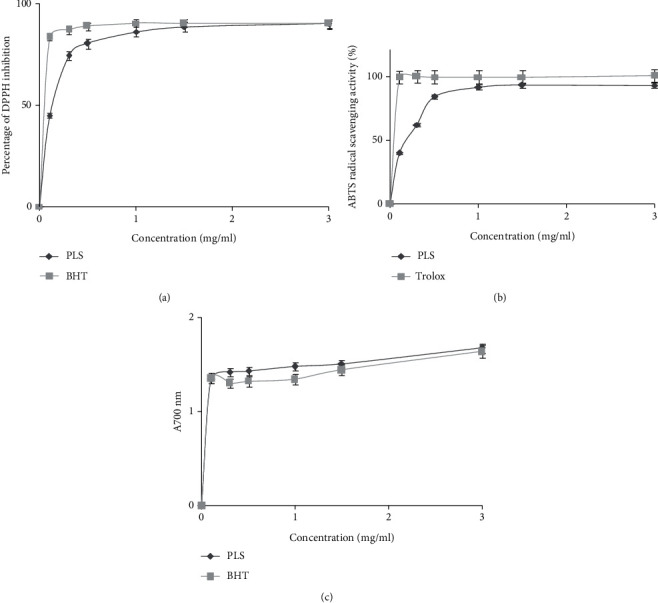
Antioxidants activities: (a) DPPH radical scavenging activity, (b) ABTS scavenging activity, and (c) reducing power determination.

As displayed in Figure 7(a), the DPPH scavenging activities increased proportionally with the rise of PLS concentrations from 0 to 3 mg/mL. At high concentration (3 mg/mL), the scavenging activities of PLS against DPPH reached 90%, which were relatively similar to those of BHT at the same dose. Furthermore, the recorded EC 50% (efficient concentration leading to 50% of activity) of PLS was 0.15 mg/mL, which was lower than that of Sorghum polysaccharides (8.5 mg/mL) [21]. This potential antioxidant activity of PLS might be due to the variety of monosaccharide compositions. Previous studies have reported that the antioxidant activities of polysaccharides could be related to different monosaccharide component ratios, among which xylose and galactose can be mentioned [38]. Furthermore, according to Feki et al. [39], the antioxidant activities of polysaccharides could be attributed due to their hydrogen donating ability, natural bioactive compounds, and functional groups.

#### 3.3.2. ABTS Radical Scavenging Activity

ABTS assay was used to transfer electrons or hydrogen atoms to inactivate this radical cation. As shown in Figure 7(b), PLS exhibits an important scavenging activity against ABTS radical in a concentration-dependent manner. The positive control BHT exhibited a high activity in comparison with the polysaccharide. PLS has a value of 99.87% at 3 mg/mL, which could relate the structures of the polysaccharides to their antioxidant activity [21]. In fact, the PLS radical-scavenging ability could be attributed to the existence of carboxyl group and hydroxyl groups in crude polysaccharides, which can act as free radical quenching [30].

#### 3.3.3. Reducing Power

The capacity of PLS to reduce the oxidation of Fe^3+^ in ferric chloride to the ferrous form of Fe^2+^ by antioxidants was investigated. As presented in Figure 7(c), the reducing power increased proportionally with PLS doses reaching a maximum (*A*700 = 1.68) at 3 mg/mL. This reducing power was higher than polysaccharide from Sorghum bicolor [6]. Parhat et al. [40] have reported that the antioxidant activities are associated with molecular weight, glycoside bond types, and configuration of polysaccharides.

### 3.4. In Vivo Wound Healing Activity of PLS

#### 3.4.1. Macroscopic Study of Wounds

**Figure 8:**
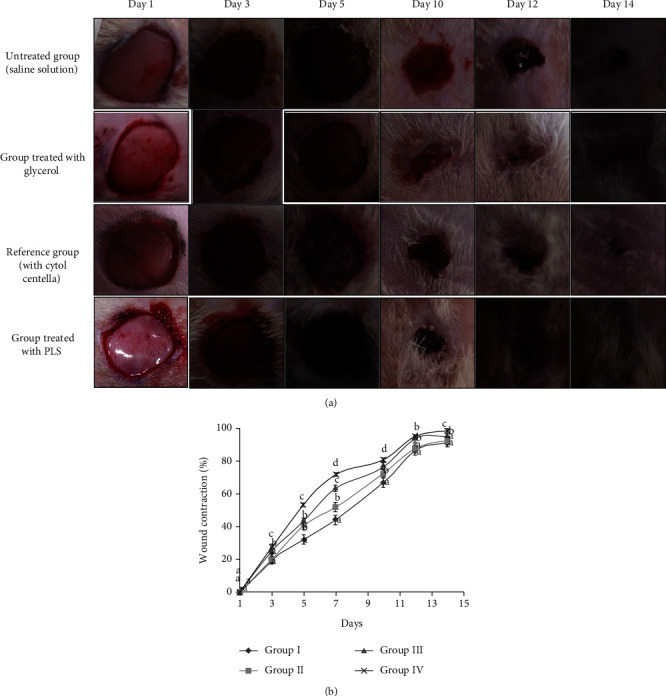
(a) Injury wounds taken for the different groups on days 1, 3, 5, 10, 12, and 14. Group I: control diabetic group treated with a saline solution; group II: diabetic group treated with glycerol; group III: positive group in which animals were treated with Cytol Centella cream; and group IV: diabetic group treated with PLS hydrogel. (b) Percentage of wound contraction for different groups. Group I: control diabetic group treated with a saline solution; group II: diabetic group treated with glycerol; group III: positive group in which animals were treated with Cytol Centella cream; and group IV: diabetic group treated with PLS hydrogel. Different letters represent significant difference at *p* < 0.05.

To investigate the wound healing promoting potential, the appearance of the wounds of the four groups of diabetic rats was continuously observed for 14 days (Figure 8(a)). On the first day, all wounds in diabetic animals demonstrated a similar appearance of a bright red color spot, which reflected blood covering into the underlying muscle after skin lesions. For the groups treated with saline solution and glycerol, crusts were observed on day 12 from injury. However, a brown color of the scabs was noticed in the “Cytol Centella” treated group (group 3) and PLS group (group 4), on days 10 and 5, respectively. A pink color of the scab falling and a complete wound closure occurred in the 14 days for group 4. Indeed, this group presented a better aspect of the wound scar and homogeneity in the wound retraction index, which reflects wound reparations and granulation tissue formation. The diabetic rats treated with PLS healed faster compared to rats in the other groups at the end of the experiment.

Such results are in line with those obtained by Mapoung et al. [41] where natural compounds exhibited the greater wound healing effects against injury tissue. These results could be related to the effect of crude polysaccharides to protect the skin from harmful factors due to their antioxidant and antiaging actions [39]. Polysaccharides have paid much interest in pharmacology as anti-inflammatory and antioxidant agents and are mainly applied due to their nontoxic component compared with synthetic ones [39]. Generally, polysaccharides have the potentiality to regulate or inhibit the migration of moisture, carbon dioxide, oxygen, and water vapor. In addition, PLS was a compatible component with the user's skin. This biocompatibility has already been approved by the allergy skin test on rats which revealed an absence of allergenic reactions and irritation. However, polysaccharides with heterogeneous complex structures may provoke the immune system to overreact and cause irritation. Consequently, the control of the molecular weight of polysaccharide is expected to overcome this limitation [9].

#### 3.4.2. Wound Closure and Epithelialization Evaluation

The rate of wound healing in diabetic rats was regularly captured for the 14-day experimental period to authenticate the property of wound healing.

The wound closure was traced, and the percentage wound contraction was calculated planimetrically (Figure 8(b)).

Diabetic groups treated with glycerol and saline solution groups showed 72.67 and 66.96% of wound contraction, respectively, on day 10. However, diabetic groups treated with PLS and Cytol Centella revealed an increased wound contraction rate on day 10 (81.39 and 76.51%, respectively), compared to control wounds. At the end of the experiment, the wound contraction in diabetic rats treated with PLS was around 98%. This result agrees with many studies in which the diabetic wounds of the untreated group always showed incomplete wound contraction than those treated with the tested healing principle [40].

Overall, our results have suggested that PLS significantly enhances wound healing in alloxan-induced diabetic rats, due to its ability to activate immune system by activating macrophages that clean up the wound site after injury. In fact, PLS application reduced the infections and accelerated the epithelization and cell proliferations. PLS hydrogel accelerated the wound healing due to its biocompatibility, nontoxicity, and high absorption capacity. However, biopolymers are commonly limited by their poor mechanical properties. They are associated with synthetic polymers in order to enhance their mechanical properties [42].

Conversely, the glycerol-treated wounds healed more slowly. This finding is in accordance with those obtained in previous research, where it has been reported that the Morin-incorporated polysaccharide-protein hydrogel accelerates diabetic wound healing in Wistar rats [39]. In addition, Li et al. [43] demonstrated that polysaccharides extracted from Dendrobium candidum could be a potential candidate as wound healing-accelerating agents thanks to their incredible wound healing properties in both in vitro and in vivo experiments.

#### 3.4.3. Histological Study

**Figure 9:**
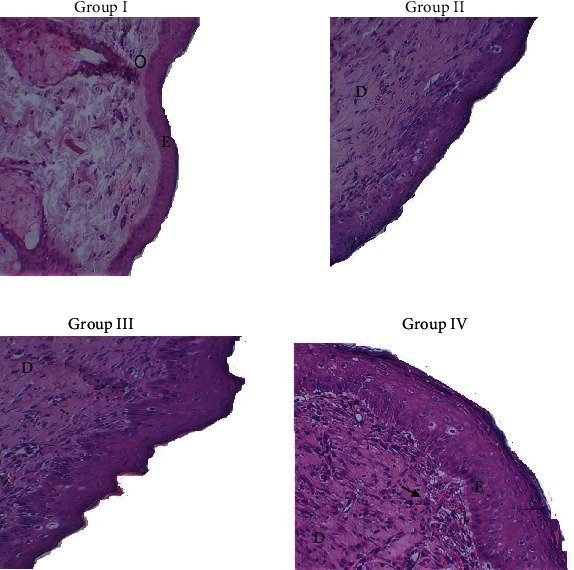
Photomicrographs of skin sections coloured with hematoxylin-eosin after 14 days of injury induction. Group I : rats treated with a saline solution; group II: diabetic rats treated with glycerol; group III: positive group in which rats were treated with Cytol Centella cream; and group IV: diabetic rats treated with PLS hydrogel. E: epidermis; D: dermis. Three independent experiments on three animals per group were used.

The histological study was performed on the 14^th^ day after injury inductions. The micrograph sections of wound healing of all experiments are shown in Figure 9.

The histological observation of the wound tissue revealed a fully epithelial regeneration with a well-structured layer with a few cells' inflammation in the PLS-treated group compared to those of the other groups. This could be related to the collagen deposition and angiogenesis at the diabetic wound site, with decreased neutrophil infiltration and enhanced blood circulation that provide more essential nutrients and oxygen for the healing process [44]. This faster wound healing of PLS could be attributed to its potential activity against intrinsic free radical scavenging activity and reactive oxygen species, as previously demonstrated using other plant polysaccharide extracts on wound healing activity [1, 27]. Indeed, these statements support our results as PLS was found to exhibit strong antioxidant activities that may have valuable effects on balancing the anti-inflammatory mediators of the local wound environment [41]. However, the histological evaluation of the diabetic wound healing in the untreated group and the group treated with glycerol showed that they differed from the other groups. In fact, the histological form of the epidermis in the group treated with glycerol and the untreated group showed the active inflammatory lesion persistence with the excess infiltration of various inflammatory cells. Moreover, group I and group II indicated an incomplete regeneration and hyperemia of capillary blood vessels. Recently, polysaccharide-based hydrogels have been widely used as drug carriers. In our study, PLS has been demonstrated as a potential cream carrier thanks to its biological properties, biocompatibility, and nontoxicity.

#### 3.4.4. Hydroxyproline Estimation

Hydroxyproline was used as a biochemical specific marker for tissue collagen stability, and thus for the estimation of collagen rate synthesis. As shown in Table 2, hydroxyproline concentrations were found to be high in the groups of rats treated with PLS polysaccharides (21.99 mg/g of tissue) compared to the control and glycerol conforming our previous results pertaining to the deposition and organization of collagen fibers in the histological study.

The significant increase in the hydroxyproline level in the PLS-treated group implies more collagen deposition and supports the polymer efficiency in fibroblastic proliferation and production of the extracellular matrix during the wound healing process. Additionally, PLS is able to react or deactivate some radicals and oxidants, which could help control wound oxidative stress, and thus accelerate wound healing [39]. In fact, in vivo, collagen synthesis in human skin is stimulated by locally applying natural polysaccharides [45].

## 4. Conclusion

This research work has reported that heteropolysaccharide, named PLS, exhibited in vitro antioxidant activities and proven to exert wound healing effects in diabetic rats due to its outstanding properties. Accordingly, PLS hydrogel had a significant function in the management of wound recovery and improved the healing process when compared with the control and reference groups. Further biochemical investigations are required to develop the accurate mechanism of this polysaccharide during wound healing effect.

## Figures and Tables

**Table 1:** Physicochemical properties of PLS.

Parameters	PLS
Yield (%)	8.2 ± 0.99
Moisture (%)	4.33 ± 0.10
Ash (%)	5.99 ± 0.65
Proteins (%)	1.23 ± 0.14
Fat (%)	0.03 ± 0.01
Polysaccharides (%)	88.58 ± 0.06
Color	
*a*∗	5.87 ± 0.01
*b*∗	27.28 ± 0.02
*L*∗ pH	63.26 ± 0.01 7.2 ± 0.02

**Table 2:** Hydroxyproline content in the tissue of the different experimental animal groups.

Groups	Hydroxyproline amounts (mg/g of tissue)
Group I	13.02 ± 2.02^a^
Group II	13.89 ± 3.05^a^
Group III	20.54 ± 2.11^b^
Group IV	21.99 ± 2.99^b^

Group I: control diabetic group treated with a saline solution; group II: diabetic group treated with glycerol; group III: positive group in which animals were treated with Cytol Centella cream; and group IV: diabetic group treated with PLS hydrogel. Values are given as **mean** ± **SD**. Data with different letters for each column represent significant difference at **p** < 0.05.

## Data Availability

No data were used to support this study.
